# Impact of Peripheral Angioplasty on Wound Oxygenation and Healing in Patients with Chronic Limb-Threatening Ischemia Measured by Near-Infrared Spectroscopy

**DOI:** 10.3390/biomedicines12081805

**Published:** 2024-08-08

**Authors:** Johanna Schremmer, Manuel Stern, Sven Baasen, Patricia Wischmann, Ramy Foerster, Miriam Schillings, Kálmán Bódis, Roberto Sansone, Christian Heiss, Malte Kelm, Lucas Busch

**Affiliations:** 1 Department of Cardiology, Pulmonology and Vascular Medicine, Medical Faculty, Heinrich Heine University, 40225 Duesseldorf, Germany; johannamaria.schremmer@med.uni-duesseldorf.de (J.S.); hanslucas.busch@med.uni-duesseldorf.de (L.B.); 2 Department of Endocrinology and Diabetology, Medical Faculty, Heinrich Heine University, 40225 Duesseldorf, Germany; 3 Institute for Clinical Diabetology, German Diabetes Center, Leibniz Center for Diabetes Research at Heinrich Heine University, 40225 Duesseldorf, Germany; 4 German Center for Diabetes Research, Partner Duesseldorf, 85764 München-Neuherberg, Germany; 5 Department of Clinical and Experimental Medicine, Faculty of Health and Medical Sciences, University of Surrey, Guildford GU2 7XH, UK; c.heiss@surrey.ac.uk; 6 Department of Vascular Medicine, Surrey and Sussex NHS Healthcare Trust, Redhill RH1 5RH, UK; 7 Cardiovascular Research Institute Duesseldorf (CARID), 40225 Duesseldorf, Germany

**Keywords:** peripheral arterial disease, chronic limb-threatening ischemia, percutaneous transluminal angioplasty, near-infrared spectroscopy, tissue oxygen saturation, wound healing

## Abstract

Managing chronic limb-threatening ischemia (CLTI) is challenging due to difficulties in assessing tissue oxygen saturation in ulcers. Near-infrared spectroscopy (NIRS) is a non-invasive method for measuring tissue oxygen saturation (StO_2_). This study evaluated the effects of endovascular treatment (EVT) on StO_2_ and wound healing in CLTI patients, comparing NIRS to standard ankle–brachial index (ABI) measurements. Using the Duesseldorf PTA Registry, 43 CLTI patients were analyzed: 27 underwent EVT, and 16 received conservative treatment. ABI assessed macrocirculation, while NIRS measured wound, wound area, and mean foot StO_2_ at baseline, post-EVT, and four-month follow-up. Wound severity was classified by wound area and wound, ischemia, and foot infection (WIfI) score. Wound StO_2_ increased significantly (median (interquartile range (IQR)), 38 (49.3) to 60 (34.5)%, *p* = 0.004), as did wound area StO_2_ (median (IQR), 70.9 (21.6) to 72.8 (18.3)%, *p* < 0.001), with no significant changes in the control group by four-month follow-up. Wound area decreased significantly after EVT (mean ± SD, 343.1 ± 267.8 to 178.1 ± 268.5 mm^2^, *p* = 0.01) but not in the control group. Changes in wound StO_2_, wound area StO_2_, and WIfI score correlated with wound area reduction, unlike ABI. This small exploratory study shows that NIRS-measured StO_2_ improvements after EVT correlate with reduced wound area and WIfI scores, highlighting NIRS as a potential enhancement for CLTI wound management in addition to ABI.

## 1. Introduction

Peripheral artery disease (PAD) is the third leading cause of atherosclerotic cardiovascular morbidity worldwide, after coronary heart disease and stroke, and is associated with an increased risk of major cardiovascular events and mortality [1]. The most severe clinical manifestation of PAD is chronic limb-threatening ischemia (CLTI). CLTI is associated with high mortality, amputation rates, and impaired quality of life [2]. Patients with CLTI present with ischemic rest pain, gangrene, or lower extremity wounds (ulcers) lasting more than two weeks [3]. The ankle–brachial index (ABI) is the primary non-invasive screening tool for PAD, with an ABI ≤ 0.90 having a positive predictive value of ≥95% for detecting PAD and correlating with its severity [4]. However, ABI is unreliable in certain populations, such as those with diabetes or chronic kidney disease, due to medial calcification, which can produce falsely high ABI values despite the presence of PAD [5,6,7,8]. In an observational study, post-intervention ABI in CLTI patients undergoing endovascular treatment was not associated with wound healing [9]. Furthermore, a meta-analysis by Wang et al. demonstrated that there is no correlation between ABI changes and wound healing in CLTI patients with diabetes [10]. This indicates that ABI is an unreliable predictor of wound healing in these patients. Color-coded duplex sonography can assess the localization and severity of arterial stenoses but is limited to medium to large vessels and cannot assess the microcirculation of the foot. The role of microcirculation in ulcer healing in CLTI patients, particularly in relation to therapeutic implications, is the subject of ongoing research and is currently still unclear [11]. There is evidence that impaired microcirculatory function is a systemic disease causing a variety of clinical manifestations such as renal failure, stroke, ischemic heart disease, or pulmonary arterial hypertension [12]. Transcutaneous oxygen pressure (TcPO_2_) and peripheral oscillography are useful in patients with unreliable ABI, such as those with media sclerosis, and for estimating wound healing capacity after revascularization [13,14]. However, these methods do not directly assess blood flow or oxygen saturation in the wound or surrounding tissue. Therefore, an improvement in wound perfusion can only be assessed indirectly. Near-infrared spectroscopy (NIRS) using a camera (e.g., SnapshotNIR, Kent Imaging©) is a relatively new, non-invasive, and non-contact method to quantify peripheral microcirculation, even within the wound. We hypothesize that NIRS measurement of microvascular tissue perfusion is a reliable and practical method for estimating wound healing potential in CLTI patients after endovascular treatment (EVT) and can predict wound area changes better than ABI measurements. 

## 2. Materials and Methods

Using the prospectively maintained “all-comers” Duesseldorf PTA Registry (Clinical Trial Registration—Unique identifier: NCT02728479), we analyzed the outcomes of 43 CLTI patients. This study was conducted according to the guidelines of the Declaration of Helsinki and approved by the Ethics Committee of Heinrich-Heine University Duesseldorf (NCT02728479). Informed consent was obtained from all subjects included in this study.

### 2.1. Patients

A total of 27 subjects underwent EVT, and 16 subjects received optimal medical therapy and wound care only (control group). The decision between EVT and conservative treatment was made on an individual basis. In most cases, allocation to the conservative group was based on individual patient preference, especially in cases where EVT was refused at the time of presentation. In the remaining cases, the treating angiologist made the treatment decision based on individual patient factors. All patients were treated in the vascular unit of the Department of Cardiology, Pneumology, and Vascular Medicine at the University Hospital Duesseldorf. Inclusion criteria were patients over 18 years of age, PAD Fontaine stage 4, presence of a peripheral arterial wound, and consent for participation.

### 2.2. Study Design

The EVT group received revascularization and optimal wound care. The control group received optimal wound care only. Patients were evaluated at the same time intervals. Vascular assessment was performed at baseline and at four months, with an additional assessment in the EVT group on the day after EVT. All examinations were performed in the outpatient unit of the vascular department. Patients were placed in the supine position for at least 10 min in a quiet, air-conditioned room (22 °C). 

### 2.3. Measurements

Office measurements were performed, including a standardized vascular ultrasound assessment (10 MHz transducer; Vivid I, GE, San Jose, United States) and measurement of the ABI. Blood pressure (BP) was measured using an automated clinical digital sphygmomanometer (Dynamap Vital Signs Monitor, Dinamap, General Electric Health Care, Solingen, Germany). ABI was calculated for the leg with the wound as the ratio of the highest lower limb blood pressure to the higher brachial systolic blood pressure, as recommended in the ESC PAD guideline [15]. StO_2_, as a surrogate parameter for microcirculatory tissue perfusion, was measured using a near-infrared spectroscopy (NIRS) device (SnapshotNIR, Kent Imaging, Canada). This type of NIRS measurement has good reproducibility and agreement with established techniques. Its current clinical use is mainly in reconstructive surgery and wound care [16,17]. This camera is capable of non-invasively detecting oxyhemoglobin/myoglobin saturation levels up to a depth of 5 mm. StO_2_ is reported as color-coded StO_2_ maps. Measurements were taken in three defined regions: the wound, defined as the region where the skin barrier was not intact; the wound area, defined as the region immediately surrounding the wound; and finally, the mean of the whole foot, obtained by measuring the sole of the foot and the toes. Five measurements of the wound and wound area, five measurements of the sole, and one measurement of the nail fold of each toe were taken for the mean of the foot. The mean was then calculated. Wound assessment was performed by measuring the wound area in mm^2^ from the NIRS photographs taken and additionally by obtaining the wound, ischemia, and foot infection (WIfI) score at pre- and post-treatment. The WIfI score is a classification system developed by the Lower Limb Guidelines Committee of the Society for Vascular Surgery (SVS) to categorize the risk factors for amputation in CLTI patients: wound, ischemia, and foot infection. It includes a scoring system for clinical and hemodynamic assessment of the lower limb, with 0–3 points possible in each category (totaling 0–9 points) [18]. The WIfI score is an established score for wound healing tendency and the need for amputation [19].

### 2.4. Statistical Analysis

StO_2_ values and wound area parameters did not show a normal distribution, so mainly non-parametric tests were used. Continuous variables were compared using the Mann–Whitney U test. Categorical and ordinal variables are presented as absolute numbers and percentages, and statistical comparisons were made using the Wilcoxon test. ABI values showed a normal distribution, so continuous variables were compared using Student’s *t*-test. Spearman correlation was used for correlations in non-parametric data and Pearson correlation was used for parametric data. Linear regression was used to identify possible predictors for outcomes. Parametric data were presented as mean ± standard deviation, and non-parametric data were presented as median and interquartile range (IQR). Data were analyzed using GraphPad Software Prism version 9.00 and IBM SPSS software version 27.0.

## 3. Results

### 3.1. Baseline Characteristics

We prospectively analyzed 43 CLTI patients with arterial foot ulcers. A total of 27 subjects underwent EVT, and 16 subjects received optimal wound care. Follow-up was approximately four months (mean 129 days) in both groups, with five patients lost to follow-up (four EVT and one in the control group). Reasons for the loss to follow-up were death in four cases and failure to return for follow-up in one case. The mean age was 77 ± 8 years (mean ± SD) in the EVT group and 75 ± 12 years (mean ± SD) in the control group. The majority of patients were male (89% in the EVT group and 100% in the conservative group). EVT was most commonly performed below the knee (26 treated lesions) and femoropopliteal (13 treated lesions). Details of patient characteristics are presented in Table 1, and further characteristics are shown in Appendix A (Table A1 and Table A2).

There were no significant differences between the two groups regarding age, sex, medication, or comorbidities at baseline. Baseline StO_2_ measurements of the mean foot (mean (interquartile range (IQR)), 66.7 (11) vs. 80.5 (5.5) %), wound area (66.1 (28.4) vs. 70.9 (21.6) %) and wound (38 (49.3) vs. 63.1 (31.4) %) tended to be lower in the EVT group, while there was no significant statistical difference. At baseline, the wound area tended to be larger in the EVT group (mean ± SD, 343.1 ± 267.4 to 178.1 ± 268.5 mm^2^) with a wound area range of 7.1–904.7 mm^2^ in the EVT group and 23.8–710.1 mm^2^ in the control group, but this observation was below the level of significance. The ABI tended to be slightly lower in the EVT group (mean ± SD, EVT: 0.72 ± 0.21, C: 0.82 ± 0.22). However, these differences were not significant. In the EVT group, a mean volume of 54 ± 24 mL contrast agent (*n* = 25) and 100 ± 28 mL CO_2_ (*n* = 2) was used during the procedure. 

### 3.2. Response to Treatment

#### 3.2.1. Wound Area

**Figure 1:**
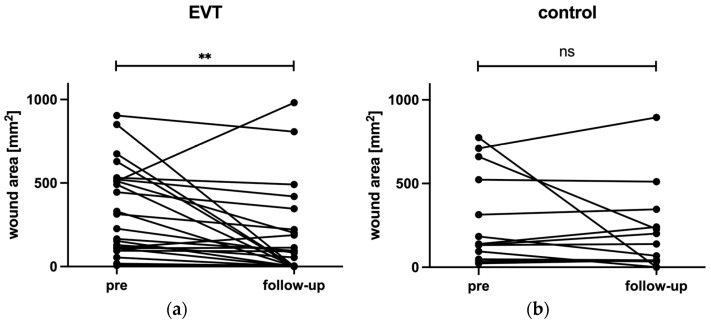
Changes in wound area between pre and four-month follow-up in the EVT group (**a**) and control group (**b**). ** = *p* < 0.01; ns = not significant. (EVT = endovascular treatment.)

At baseline, there was no significant difference in wound area between the two groups (EVT: 343.1 ± 267.8 mm^2^; C: 272.3 ± 274.1 mm^2^; *p* = 0.44). In the EVT group, there was a significant reduction in wound area between pre and follow-up (343.1 ± 267.8 mm^2^ vs. 178.1 ± 268.5 mm^2^, *p* = 0.009). In the control group, there were no significant changes in wound area between pre and follow-up (272.3 ± 274.1 mm^2^ vs. 199.2 ± 249.1 mm^2^, *p* = 0.249) (Figure 1). There was a significant difference in wound area reduction between the two groups from pre to follow-up (EVT: −165 ± 280.3%; C: −73.1 ± 245.8%; *p* = 0.032).

#### 3.2.2. WIfI Score

**Figure 2:**
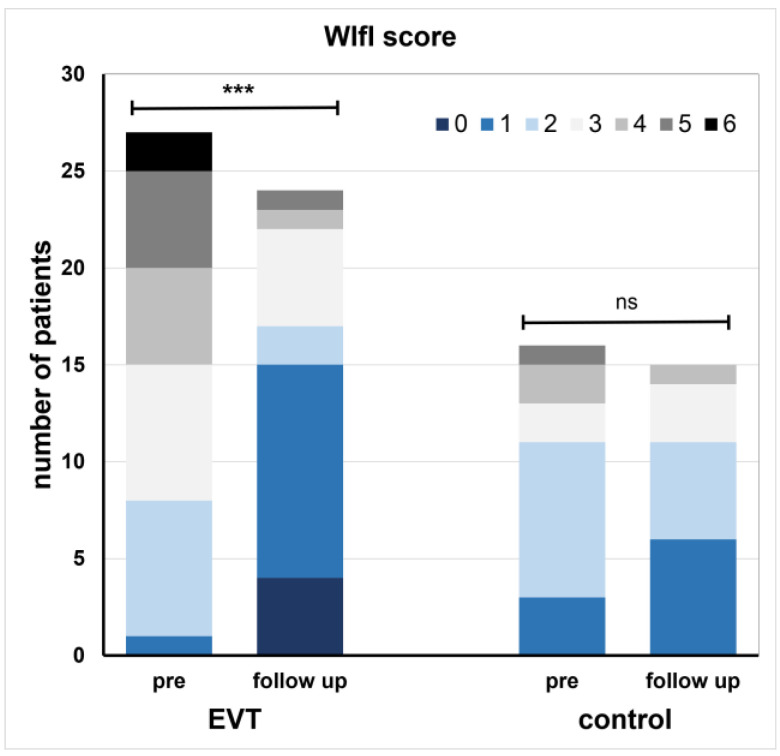
Absolute WIfI scoring of the study participants in the EVT group and the control group; max. rating in this study population was 6 out of 9 points. *** = *p* < 0.001; ns = not significant. (EVT = endovascular treatment.)

There was no significant difference in the WIfI score between the two groups at baseline (mean ± SD, EVT: 3.5 ± 1.4 points; C: 2.5 ± 1.3 points *p* = 0.29), while the WIfI score tended to be higher in the EVT group. The maximum score in the EVT group was 6 out of 9 possible points, while the maximum score in the control group was 5 points at baseline. In the EVT group, there was a significant improvement in the WIfI score between pre and follow-up (3.5 ± 1.4 points vs. 1.7 ± 1.5 points, *p* < 0.001), whereas in the control group, there was no difference between pre and follow-up (2.5 ± 1.3 points vs. 1.8 ± 1 points, *p* = 0.059) (Figure 2). The exact scoring in the EVT group between pre and follow-up was wound (W) 1.7 ± 0.6 vs. 0.83 ± 1 points, ischemia (I) 1.1 ± 0.8 vs. 0.7 ± 0.7 points, and foot infection (FI) 0.7 ± 0.7 vs. 0.2 ± 0.4 points. Exact scoring in the control group between pre and follow-up was wound (W) 1.5 ± 0.8 vs. 1.1 ± 0.8 points, ischemia (I) 0.8 ± 0.7 vs. 0.7 ± 0.7 points, and foot infection (FI) 0.2 ± 0.4 vs. 0.1 ± 0.3 points. A significant correlation was observed between changes in WIfI score and reduction in wound area in the study population (r = 0.513, *p* = 0.002). In the subgroup analysis, the correlation remained significant in the EVT group (r = 0.558, *p* = 0.016), while there was no correlation in the control group (*p* = 0.59). 

### 3.3. Prediction of Wound Healing

#### 3.3.1. NIRS Measurements

**Figure 3:**
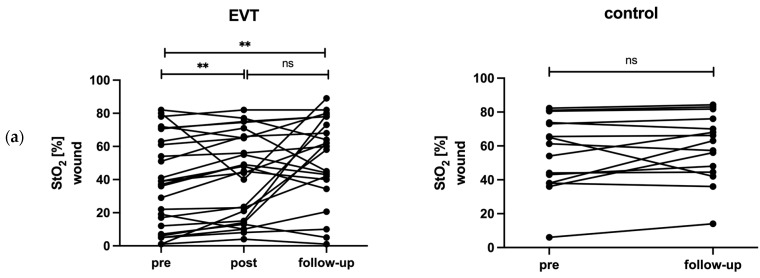

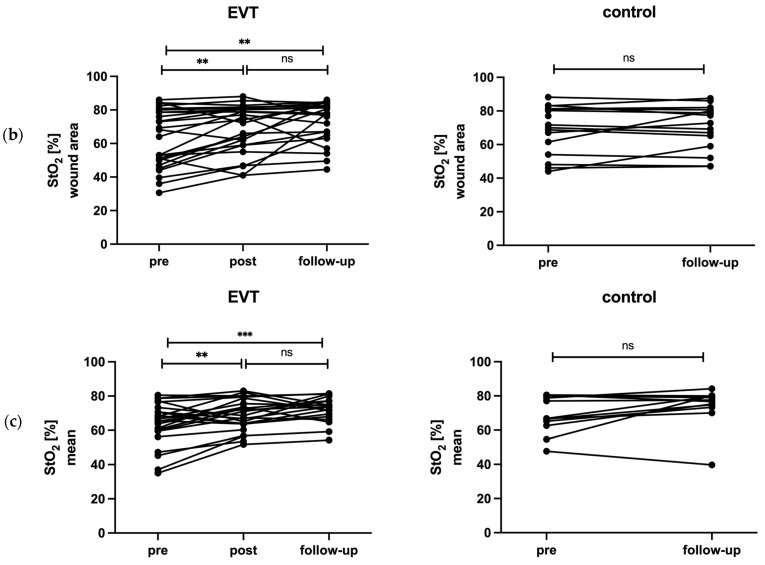
StO_2_ of the wound (**a**), wound area (**b**), and mean foot (**c**) between pre and four-month follow-up. ** = *p* < 0.01; *** = *p* < 0.001; ns = not significant. (EVT = endovascular treatment, StO_2_ = tissue oxygen saturation.)

The EVT group showed a significant increase between pre- and post-intervention in median StO_2_ values of the wound (median (IQR), pre 38 (49.3)% vs. post 45 (50.0)%, *p* = 0.01), the wound area (pre 66.1 (28.4)% vs. post 72.2 (21.1)%, *p* = 0.002) and the mean foot (pre 66.7 (11.0)% vs. post 69.8 (11.9)%, *p* = 0.005). Concurrently, StO_2_ values increased significantly between pre and follow-up measurements of the wound (38 (49.3)% vs. 60 (34.5)%, *p* = 0.004), the wound area (66.1 (28.4)% vs. 78 (16.8)%, *p* < 0.001) and the mean foot (66.7 (11)% vs. 73.8 (7.7)%, *p* < 0.001). In the control group, there were no significant changes between pre and follow-up in StO_2_ values of the wound (63.1 (41.8)% vs. 63 (26.8)%, *p* = 0.17), the wound area (70.9 (21.6)% vs. 72.8 (18.3)%, *p* = 0.89) and the mean foot (66.8 (14.6)% vs. 77 (15.4)%, *p* = 0.084). The data are shown in Figure 3.

**Figure 4:**
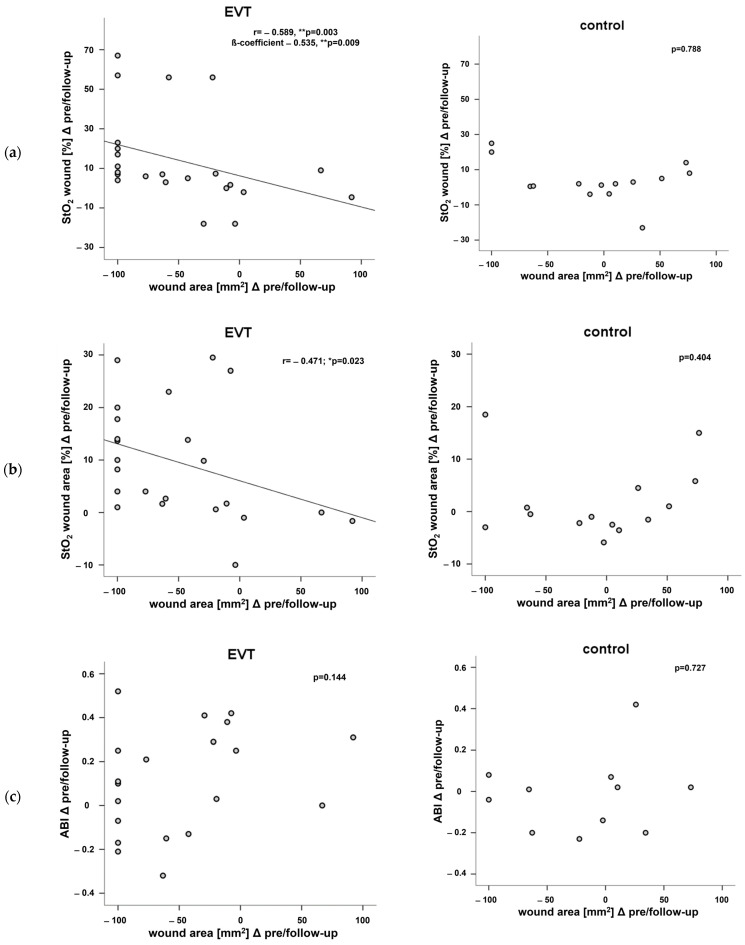
Correlations between changes in wound area and changes in StO_2_ of the wound (**a**), wound area (**b**), and ABI (**c**) between pre and follow-up in the EVT and the control group. ** = *p* < 0.01; * = *p* < 0.05. (ABI = ankle–brachial index, EVT = endovascular treatment, StO_2_ = tissue oxygen saturation.)

**Figure 5:**
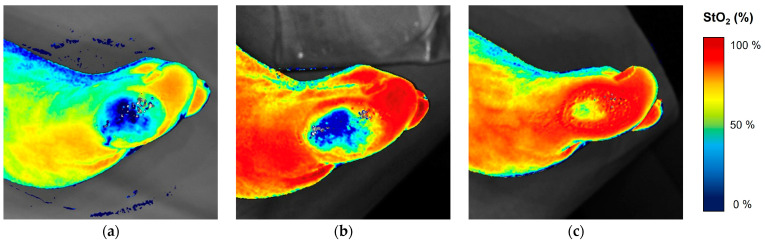
Example of NIRS camera images of a left foot ulcer (digitus 1) before (**a**), after (**b**), and at four-month follow-up (**c**) after endovascular treatment. (StO_2_ = tissue oxygen saturation.)

The reduction in wound area from baseline to follow-up correlated significantly with improvements in wound StO_2_ (r = −0.47, *p* = 0.004) and wound area StO_2_ (r = −0.395, *p* = 0.015) in the study population (Figure 4). In the subgroup analysis, the correlation was also significant in the EVT group (wound: r = −0.579, *p* = 0.004, wound area: r = −0.459, *p* = 0.028), whereas the correlation remained below the level of significance in the control group (wound: *p* = 0.668, wound area: *p* = 0.237). Linear regression revealed that StO_2_ changes in the wound, but not the wound area, predicted a reduction in wound area (β-coefficient −0.535, *p* = 0.009; see Figure 4), unlike the control group in the observed study population. Age, smoking, and diabetes were not predictors of changes in wound area. Changes in WIfI score significantly correlated with changes in wound StO_2_ (r = −0.428, *p* = 0.042) between pre- and post-intervention. In addition, changes in WIfI score correlated significantly with changes in StO2 of the wound (r = −0.419, *p* = 0.011) and wound area (r = −0.390, *p* = 0.019) between pre and follow-up. These results remained below the significance level when the two groups were analyzed separately. Figure 5 shows an example of the different NIRS photos over time in one patient after EVT.

#### 3.3.2. ABI Measurements

**Figure 6:**
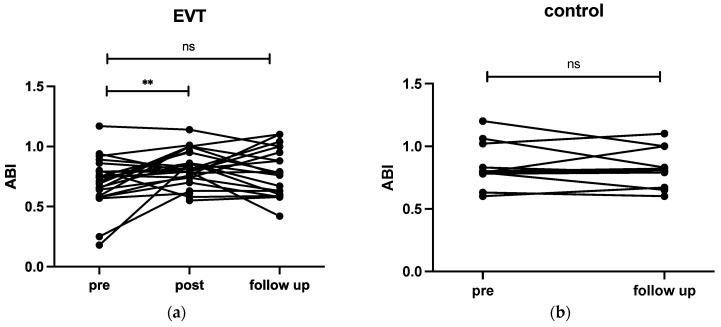
Ankle–brachial index (ABI) of the affected leg between pre and follow-up in the EVT (**a**) and the control group (**b**). ** = *p* < 0.01; ns = not significant. (EVT = endovascular treatment.)

The EVT group showed a significant increase in ABI between pre- and post-intervention (mean ± SD, 0.72 ± 0.21 vs. 0.83 ± 0.15, *p* = 0.005), but no significant increase between pre and follow-up (0.72 ± 0.21 vs. 0.8 ± 0.2, *p* = 0.061). In the control group, there were no significant changes in ABI values of the affected leg between pre and follow-up (0.82 ± 0.23 vs. 0.84 ± 0.16, *p* = 0.98) (Figure 6). There were no correlations between ABI measurements and changes in StO_2_, changes in wound area, or WIfI score in both groups. ABI was not a predictor of changes in wound area.

## 4. Discussion

This study demonstrates for the first time that (i) successful EVT in CLTI patients improves wound and wound area tissue oxygen saturation, and (ii) improvement in wound and wound area tissue oxygen saturation correlates with wound healing according to wound area and WIfI score, unlike the ABI. This suggests that advanced arterial wound monitoring with NIRS may improve the management of arterial wounds in CLTI patients. 

### 4.1. Management of CLTI Patients

CLTI patients with chronic ulcers often require endovascular or surgical treatment, but standard technologies (ABI, duplex ultrasound) cannot reliably assess early treatment efficacy. Few studies have evaluated diagnostic tools for wound healing in CLTI patients. Kayama et al. used a finger-mounted tissue oximeter in 34 CLTI patients with foot ulcers and found that a tissue oxygen saturation threshold of ≥50% predicted wound healing [20]. Maheshwari et al. analyzed 14 PAD patients with chronic wounds after revascularization using the Dynamic Vascular Optical Spectroscopy (DVOS) system, showing a strong correlation between a hemoglobin parameter and wound healing [21]. Other studies have evaluated the peripheral microcirculation in PAD patients after revascularization without focusing on wound healing. Geskin et al. used the same NIRS camera as in this study to evaluate tissue perfusion in patients with PAD without ulcers before and after revascularization and found a significant increase in StO_2_ values after intervention but no correlation with changes in ABI. These results are consistent with our findings and support the hypothesis that ABI is not a reliable marker of peripheral tissue perfusion after revascularization [22]. Boezeman et al. used a single optode near foot ulcers to measure StO_2_ and also found significant increases after revascularization [23]. These studies did not measure directly in the wound, as the tools used were not designed for this, highlighting the differences in global and local oxygen saturation that influence wound healing. This study is the first to evaluate tissue oxygen saturation in the wound and surrounding area as a predictor of wound healing before and after EVT in CLTI patients.

### 4.2. Improvement in Wound Tissue Oxygenation Correlates with Wound Healing 

This small, prospective exploratory study shows significant increases in StO_2_ values in the whole foot, the wound area, and the wound itself after EVT, with no significant changes in the control group at four months follow-up. These results were expected due to the revascularization of flow-limiting stenoses. In addition, a correlation was observed between the improvement in StO_2_ values of the wound and wound area, which can be considered a surrogate parameter for microcirculation, and the reduction in wound area in the study population. Based on these findings, it can be discussed whether the wound area may be used as an absolute surrogate parameter for ischemia in PAD patients. Wound healing in PAD patients with foot ulcers is complex and multifaceted, requiring a multidisciplinary approach. It depends on several factors, including adequate blood supply, infection control, wound debridement, and patient compliance. Therefore, wound area was not considered as an absolute surrogate parameter in this study as it does not adequately represent the complexity of wounds. In the EVT group, there were no significant changes between post-EVT and follow-up, nor was wound StO_2_ at post-EVT a predictor of wound healing. A possible explanation for this observation could be a transient contrast medium effect after angiography, as the post-EVT measurements were taken the day after EVT. This hypothesis is supported by the fact that some patients initially showed a reduction in StO_2_ post-EVT, whereas StO_2_ increased again at follow-up (see Figure 3). However, no correlation was found between the ABI, the current gold standard in the diagnosis of PAD, and the peripheral StO_2_ values before or after EVT. Changes in ABI and StO_2_ showed no correlation. The results suggest that the ABI and tissue oxygen saturation are parameters to be considered and evaluated independently. These findings are consistent with previous studies [22,23,24] and demonstrate the importance of considering local wound tissue oxygenation. 

### 4.3. NIRS Monitoring of Complex Arterial Wounds

A significant advantage of NIRS is its ability to quantify tissue oxygen saturation directly in and around the wound in a non-invasive and non-contact manner. This small, prospective exploratory study indicates that NIRS is a rapid, efficient, and practical method for assessing surrogate parameters of wound perfusion and estimating healing tendencies in CLTI patients post-EVT. NIRS is particularly reliable for low-risk wounds with small or no areas of gangrene/necrosis, which are common in clinical practice (mean WIfI score 3.4 at baseline in the EVT group, maximum WIfI score 6). The correlation between changes in wound area as well as WIfI score and StO_2_, but not ABI, supports the hypothesis that ABI is not a reliable parameter for estimating wound healing in CLTI patients. A possible explanation for why the correlation between StO_2_ and wound area in the subgroup analysis was significant only in the EVT group but not in the control group could be the small number of patients in the control group. Changes in wound StO_2_ between pre and follow-up were a predictor of wound area reduction. For a better clinical assessment of the wound healing tendency immediately after EVT, it would be useful to know whether StO_2_ changes between pre- and post-EVT are also a predictor of wound healing. In our study population, the post-EVT measurements were taken on the day after the EVT, which may have been too early to measure significant differences, as other factors (e.g., use of contrast medium on the previous day, time to final improvement in tissue oxygen saturation) may have confounded the results.

### 4.4. Limitations

The limitations of this study include the small study population and the lack of randomization. The limited number of participants may affect the statistical power, generalizability, and reproducibility of this study. This study was not randomized, which may have biased the results in this cohort. There were small differences between the groups at baseline, but these were below the level of significance. The EVT group had lower tissue oxygen saturation, lower ABI, and larger wounds. These circumstances may have influenced the decision of the treating vascular specialist as to when to perform revascularization and which group to allocate the patient to. There was a loss to follow-up of five in the EVT group and one in the control group. Some data could not be analyzed because not all parameters could be determined in every patient. In addition, this was a predominantly male study cohort, making it difficult to extrapolate the findings to female patients with CLTI. 

## 5. Conclusions

This small exploratory study demonstrates the efficacy of EVT in CLTI patients using NIRS to monitor StO_2_ in wounds and surrounding areas, highlighting significant improvements in wound perfusion and healing outcomes after EVT. The results suggest that NIRS is a practical, rapid, and efficient method for assessing wound perfusion and healing tendencies in CLTI patients, offering advantages over the traditional ABI, which did not reliably correlate with wound healing in this study. Despite this study’s small sample size and lack of randomization, the results suggest that NIRS could improve clinical practice by better predicting healing outcomes. In conclusion, this study highlights the importance of local wound StO_2_ in predicting healing outcomes and supports the use of NIRS as a superior diagnostic tool over ABI in assessing the efficacy of revascularization procedures in CLTI patients. Future studies should aim to confirm these findings and explore the long-term benefits of incorporating NIRS into the standard care of CLTI patients.

## Figures and Tables

**Table 1:** Baseline characteristics of the study population.

	EVT (*n* = 27)	Control (*n* = 16)	*p*-Value
Patient characteristics			
Male sex	24 (88.9)	16 (100)	0.21
Age—year(s)	77 ± 8	75 ± 12	0.55
Mean follow-up time—day(s)	129 ± 32	129 ± 47	0.40
Body mass index—kg/m^2^	28 ± 6	28 ± 5	0.88
Hypercholesterolemia	14 (52)	9 (56)	0.78
Hypertension	26 (96)	15 (94)	0.71
Coronary artery disease	21 (78)	13 (81)	0.79
Peripheral artery disease IV	27 (100)	16 (100)	1.0
Diabetes mellitus	22 (81)	13 (81)	0.99
Chronic kidney failure	23 (85)	12 (75)	0.62
Dialysis	2 (7)	1 (6)	0.89
Smoker	23 (85)	15 (94)	0.40
Laboratory			
Hemoglobin—g/dL	11.8 ± 2	11.9 ± 2	0.87
Creatinine—mg/dL	1.9 ± 1.5	1.7 ± 1.1	0.51
LDL cholesterol—mg/dL	87 ± 23	91 ± 21	0.64
HbA1c—%	7.3 ± 1.7	7 ± 2	0.59
Peripheral hemodynamics			
Systolic blood pressure—mmHg	142	143	0.97
Diastolic blood pressure—mmHg	76	71	0.71
ABI	0.72 ± 0.21	0.82 ± 0.22	0.34
Wound characteristics baseline			
Mean foot—% StO_2_	66.7 (11)	80.5 (5.5)	0.49
Wound area—% StO_2_	66.1 (28.4)	70.9 (21.6)	0.45
Wound—% StO_2_	38 (49.3)	63.1 (31.4)	0.12
Wound area—mm^2^	343.1 ± 267.4	272.3 ± 274.1	0.44
Wound closed by follow-up	9 (33)	2 (13)	0.66
WIfI score	3.5 ± 1.4	2.5 ± 1.3	0.29

Data are presented as *n* (%), mean ± standard deviation, or median (interquartile range). (ABI = ankle–brachial index, EVT = endovascular treatment, StO_2_ = tissue oxygen saturation, WIfI score = wound, ischemia, and foot infection score.).

## Data Availability

The data that support the findings of this study are available upon request from the corresponding author. The data are not publicly available due to privacy or ethical restrictions.

## Abbreviations

ABI = ankle–brachial index, ACE inhibitor = angiotensin-converting-enzyme inhibitor, ASS = aspirin, AT1 antagonist = angiotensin 1 receptor antagonist, BP = blood pressure, BTK = below the knee, CLTI = chronic limb-threating ischemia, e.g., = example given, EVT = endovascular treatment, IQR = interquartile range, NIRS = near-infrared spectroscopy, NOACs = non-vitamin K antagonist oral anticoagulants, PAD = peripheral artery disease, PTA = percutaneous transluminal angioplasty, SD = standard deviation, StO_2_ = tissue oxygen saturation, SVS = Society for Vascular Surgery, TcPO_2_ = transcutaneous oxygen pressure, WIfI score = wound, ischemia, foot infection score.

## Appendix A

**Table A1:** Medication of the study population.

Medication	EVT (*n* = 27)	Control (*n* = 16)	*p*-Value
ASS	13 (48)	9 (56)	0.61
P2Y12 inhibitor	12 (44)	6 (38)	0.66
NOACs	11 (41)	5 (31)	0.54
Cumarine	9 (33)	3 (19)	0.31
Statin	21 (78)	15 (94)	0.18
Metformin	8 (30)	4 (25)	0.75
Insulin	16 (63)	10 (63)	0.98
Betablocker	23 (85)	11 (69)	0.35
ACE inhibitor/AT1 antagonist	16 (59)	11 (69)	0.55
Calciumantagonist	10 (37)	9 (56)	0.23
Diuretics	23 (85)	12 (75)	0.31

Data are presented as *n* (%) (ACE inhibitor = angiotensin-converting-enzyme inhibitor, AT1 antagonist = angiotensin 1 receptor antagonist, ASS = aspirin, EVT = endovascular treatment, NOACs = non-vitamin K antagonist oral anticoagulants).

**Table A2:** Vessel segment of the procedure/stenosis.

Localization of Stenosis	EVT	Control
Iliacal	4	3
Femoropopliteal	13	12
Below the knee (BTK)	26	17
Bypass	1	1

Data are presented as absolute numbers (EVT = endovascular treatment).
